# The role of computed tomography in quantitative assessment of tracheal morphology before and after pulmonary artery sling surgery

**DOI:** 10.3389/fped.2026.1776257

**Published:** 2026-04-13

**Authors:** Lei Zheng, Ping Chen, Xian-Ying Zheng

**Affiliations:** 1 Department of Radiology, Fujian Children’s Hospital (Fujian Branch of Shanghai Children’s Medical Center), College of Clinical Medicine for Obstetrics & Gynecology and Pediatrics, Fujian Medical University, Fuzhou, China; 2 Department of Radiology, Clinical Oncology School of Fujian Medical University, Fujian Cancer Hospital, Fuzhou, China; 3 Department of Radiology, Fujian Maternity and Child Health Hospital, Fuzhou, China

**Keywords:** computed tomography, narrow-width ratio, pulmonary artery sling, quantitative, tracheal carina angle

## Abstract

**Objective:**

To investigate the clinical utility of computed tomography (CT) in the quantitative evaluation of pediatric tracheal morphology in the context of pulmonary artery sling surgery.

**Methods:**

A retrospective analysis was performed on 17 pediatric cases diagnosed with pulmonary artery sling at Fujian Children's Hospital between April 2021 and May 2025. CT imaging data were reconstructed and quantitatively analyzed to objectively evaluate tracheal morphological changes pre- and post-operatively.

**Results:**

Both ultrasound and CT can accurately detect pulmonary artery slings. Among the various noninvasive methods available for assessing tracheal morphology and obtaining quantitative measurements, we rely more heavily on CT. In this study, 12 of the 17 pediatric patients underwent postoperative CT follow-up. Quantitative CT assessment of the trachea in these 12 patients before and after surgery demonstrated improvement in tracheal narrowing postoperatively compared with preoperative status, as measured by both diameter and cross-sectional area. Postoperative narrow-to-wide ratios increased, and tracheal carina angles decreased. The diameter-based NWR increased from 0.41 ± 0.17 to 0.73 ± 0.12; the area-based NWR increased from 0.22  ±  0.19 to 0.52 ± 0.13; and the tracheal carina angle decreased from 120.32° ± 10.58°to 77.11° ± 10.60°. All observed differences were statistically significant (*p* < 0.001).

**Conclusions:**

CT imaging serves as a comprehensive diagnostic tool, enabling the identification of pulmonary artery slings as well as the detection of associated cardiovascular anomalies and tracheal abnormalities, thereby facilitating preoperative surgical planning. Furthermore, quantitative CT analysis of tracheal morphology both pre- and post-surgery provides a more precise and objective evaluation of tracheal changes.

## 1 Introduction

Pulmonary artery sling (PAS) represents a rare congenital vascular anomaly characterized by the aberrant origin of the left pulmonary artery from the right pulmonary artery, which subsequently courses posteriorly between the trachea and esophagus in a “sling-like” configuration before reaching the left hilum (1–3). The estimated prevalence of PAS is approximately 0.006% (4). This condition frequently coexists with complete tracheal rings, forming a “ring-sling complex” that leads to structural abnormalities of the tracheal cartilage and consequent luminal stenosis. The anomalous left pulmonary artery mechanically compresses the trachea, while concomitant tracheal cartilage dysplasia further exacerbates airway obstruction. This dual pathological mechanism contributes to severe tracheal stenosis in approximately 60%–80% of affected patients (5). Notably, the disease primarily presents with respiratory symptoms. Given its nonspecific presentation, the condition is prone to diagnostic delay or error. If treatment is delayed, the mortality rate can reach as high as 90% (6, 7). Therefore, early diagnosis and prompt treatment are critical (7).

Although CT angiography and echocardiography are both used to detect pulmonary artery slings, ultrasonography offers limited anatomic delineation of the trachea. This study aims to employ CT for a quantitative and objective evaluation of tracheal changes in pediatric patients with pulmonary artery slings, the findings confirmed significant postoperative improvement in tracheal morphology and effective relief of stenosis, thereby substantiating the fundamental principles underlying surgical management.

## 2 Methods and materials

### 2.1 General information

This retrospective study included 17children diagnosed with pulmonary artery sling by ultrasound between April 2021 and May 2025, comprising 4 males and 13 females, aged from 7 days to 47 months (median age 5 months). All patients presented with varying degrees of wheezing, cough, and respiratory tract infection; heart murmurs were detected in 10 cases. Patients with allergy to iodine contrast agents or renal insufficiency were excluded. The remaining patients underwent computed tomography angiography (CTA) prior to cardiac surgery. Among them, two pediatric patients had undergone tracheal intubation before the CTA scan, with the tip of the endotracheal tube positioned at approximately the level of the upper margin of the C7 vertebral body. Fourteen patients underwent surgical intervention, and twelve underwent follow-up chest CT scans within one month after surgery (range from 11 to 25 days; median 18.5 days). None of these patients was intubated during the CT scans. This study was approved by the Medical Ethics Committee of our hospital (approval 2025ETKLRK10003, granted October 13, 2025).

### 2.2 Instruments and methods

A GE Revolution 256-row spiral CT scanner was used. Children with poor cooperation were sedated with 10% chloral hydrate (0.25–0.50 mL/kg) before examination. For enhanced scanning, the non-ionic iodine contrast agent iopromide (300 mgI/mL) was injected via the forearm or dorsalis pedis vein at a dose of 1.2–1.5 mL/kg body weight, with a bolus flow rate of 0.9–2.3 mL/s (adjusted based on the child's vascular condition; a lower flow rate was used if vascular access was poor). The As Low As Reasonably Achievable (ALARA) principle was followed (8). Scanning parameters: tube current was set to automatic milliamps, tube rotation speed was 0.28 s/rev, and slice thickness was 0.625 mm.

Preoperative cardiac CTA was performed from the suprasternal notch to the cardiac base. The tube voltage was set at 70 kV. Prospective ECG gating was used without bed movement. The acquisition window center was set to 45% of the R-R interval, and the scan was manually triggered at the four-chamber heart level as the monitoring level. Image reconstruction was performed by adaptive statistical iterative reconstruction (ASiR-V) with a reconstruction interval of 5% (30% to 55%) of the R-R interval and a reconstruction slice thickness of 0.625 mm. Postoperative chest plain CT scan was performed from the lung apex to the costophrenic angle, with tube voltage set at 80 to 100 kV and a reconstruction slice thickness of 1.25 mm. All examinations were performed during spontaneous breathing.

### 2.3 Radiation dose assessment

The volumetric CT dose index (CTDIvol), dose length product (DLP), and effective dose (ED) were used as indicators to evaluate radiation exposure.

### 2.4 Data collection

Clinical characteristics, surgical records and imaging data were collected from the hospital's electronic medical record system and Picture Archiving and Communication System (PACS). The CT images were uploaded to the GE AW4.6 post-processing workstation for reconstruction, including Maximum Intensity Projection (MIP), Volume Rendering (VR), and Minimum Intensity Projection (MinIP), to better visualize the anatomical relationships among the heart, great vessels, and trachea. Using the medical image 3D reconstruction software *Mimics 21.0*, surrounding tissues were removed, the target image range was defined, and a 3D digital model of the trachea was reconstructed to obtain measurements of tracheal stenosis length, tracheal diameter, cross-sectional area, and tracheal carina angle.

All CT imaging data were anonymized and independently evaluated by two senior radiologists using *Mimics 21.0*. Both were blinded to the patients’ preoperative and postoperative status.

### 2.5 Main evaluation indicators

Changes in tracheal features before and after surgery were quantitatively evaluated using CT. Based on tracheobronchial morphology changes, PAS was classified into types I, II, and subtypes A, and B according to the Wells typing method (1, 9). Type I: tracheal bifurcation located at the T4-5 vertebral level; Type II: tracheal bifurcation located at T6-7 vertebral level; Subtype A: presence of a bronchial opening in the right upper lobe; Subtype B: absence of a bronchial opening in the right upper lobe. Based on above, quantitative assessment of CT images is used to evaluate the presence, length, and degree of tracheal stenosis. Three-dimensional reconstruction of the trachea is also used to identify any bridging bronchi. Preoperatively, these findings are comprehensively evaluated to guide the decision for surgical intervention. Postoperatively, quantitative analysis is performed to assess pre-to-postoperative changes.

Key indicators for assessing the degree of stenosis included the narrow-width ratio (NWR), measured by tracheal diameter or cross-sectional area. The plane affected by pulmonary artery sling (PAS) compression is identified as the narrowest plane, whereas the plane at the proximal end of the trachea, unaffected by the long-segment stenosis, is designated as the widest plane. If the narrowest diameter is in the anteroposterior dimension, the corresponding widest diameter is measured in the same orientation; similarly, if the narrowest diameter is lateral, the widest diameter is also measured laterally. The primary indicator for evaluating tracheal morphological changes is the tracheal carina angle. The tracheal carina angle was defined as the angle formed by the intersection of two lines drawn along the central long axis of the left and right main bronchi. For patients with abnormal tracheobronchial branching, the tracheal carina angle was measured at the inferior carina.

Clinical improvement is primarily defined as successful weaning from oxygen support with a transition to spontaneous breathing postoperatively, reduced wheezing and activity limitations, and significant resolution of pulmonary inflammation as confirmed by chest CT.

### 2.6 Data analysis

Perform consistency checks on data measured by two observers. For measurements with good consistency, calculate the average of the two readings. For data with moderate or poor consistency, reassess both datasets, including reviewing data distribution and identifying outliers, and analyze potential causes of discrepancies. ICC ≥ 075 indicates good consistency. Data were analyzed using SPSS software, version 27.0. Preoperative and postoperative changes in tracheal diameter or cross-sectional area narrow-to-width ratio and tracheal carina angle were primarily compared. Intergroup comparisons of enumeration data were performed using the chi-square test or Fisher's exact test. Given the small sample size, this study used the Shapiro–Wilk test to assess the normality of continuous variables. Normally distributed data were presented as mean ± standard deviation ($\bar{x}$ ± SD), and groups were compared using paired t-test. Non-normally distributed data were expressed as median, and groups were compared using *Wilcoxon* signed-rank test. *P* < 0.001 suggested a significant difference.

## 3 Results

### 3.1 General characteristics

Seventeen cases presented with respiratory symptoms, both echocardiography and cardiac CTA revealed pulmonary artery slings. Fifteen patients (88.24%) had single or multiple intracardiac abnormalities, including atrial septal defect (12 cases, 70.59%), ventricular septal defect (4 cases, 23.53%), patent ductus arteriosus (6 cases, 35.29%), persistent left superior vena cava (4 cases, 23.53%), and aberrant right subclavian artery (1 cases, 5.88%). All patients had long-segment tracheal stenosis, including bronchial bridges (5 cases, 29.41%) and unilateral pulmonary hypoplasia (3 cases, 17.65%). Some patients presented with extrathoracic deformities, including tethered spinal cord (2 cases, 11.76%), and one case (5.88%) each of laryngeal cartilage softening, polydactyly, butterfly vertebrae, and anal atresia with rectocutaneous fistula.

### 3.2 Treatment and follow-up

Seventeen patients received symptomatic treatment, including anti-infectives, anti-asthmatics, and respiratory support. Three patients did not complete treatment due to either severe illness or financial constraints. The remaining fourteen cases underwent surgical intervention: one underwent staged PAS and slide tracheoplasty, 13 underwent simultaneous PAS correction and slide tracheoplasty. Additionally, intraoperative bronchoscopy confirmed the presence of complete tracheal rings. The long constricting segment was then resected. Associated cardiac defects were corrected simultaneously in affected patients.

There was no in-hospital mortality. Postoperative improvement was observed in 12 patients (85.7%), with marked alleviation of respiratory symptoms. Following clinical stabilization, these patients were discharged. To objectively assess lung conditions and surgery-induced changes in tracheal morphology, all underwent plain chest CT prior to discharge (performed 11–25 days postoperatively; median, 18.5 days) to obtain first postoperative imaging. Telephone follow-up indicated that most patients received chest CT at least 6 months postoperatively; however, further CT data could not be obtained, as these follow-up scans were performed at other institutions. The remaining two patients (14.3%) experienced poor postoperative recovery with difficult extubation; both presented with severe pulmonary infection accompanied by pulmonary hemorrhage and multiple organ dysfunction. Additionally, one patient developed poor incisional healing, generalized edema, and persistent pulmonary hypertension, while another exhibited mixed acid-base imbalance postoperatively. This patient had a preoperative generalized rash that progressed after surgery, resulting in skin ulceration and hemorrhage. Owing to the critical condition, the family declined further treatment and requested discharge. Telephone follow-up confirmed the patient's death.

### 3.3 Tracheal characteristics and morphological changes

Seventeen children underwent preoperative cardiac CTA. Classification revealed 7 cases (41.18%) of type IA, 7 cases (41.18%) of type IIA, and 3 cases (17.64%) of type IIB. All pediatric patients exhibited long-segment tracheal stenosis, highly suggestive of the diagnosis of complete tracheal rings. Interobserver agreement was high when assessing preoperative CT images (Table 1). Consequently, the mean values of all measurements-including stenosis length, diameter, cross-sectional area, and carinal angle-were used for analysis. Results showed that preoperative NWR for tracheal diameter was 0.43 ± 0.16, preoperative NWR of tracheal cross-sectional area was 0.25 ± 0.17, and preoperative tracheal carina angle was 121.68° ± 9.96°.

**Table 1:** Interobserver and intermethod agreement on preoperative CT measurements (*n* = 17).

Parameters	Length of the narrow segment (mm)	Smallest diameter (mm)	Maximum diameter (mm)	Narrowest cross-sectional area (mm^2^)	Maximum cross-sectional area (mm^2^)	Tracheal carina angle °
Observer1	25.47 ± 11.58	2.00 ± 0.69	4.79 ± 0.90	4.70 ± 2.85	22.36 ± 11.77	119.99 ± 10.10
Observer2	28.89 ± 11.44	2.13 ± 0.76	4.96 ± 1.03	5.86 ± 3.79	26.76 ± 11.78	123.37 ± 9.95
ICC	0.978	0.952	0.958	0.973	0.997	0.973
95%CI	[0.942,0.992]	[0.873,0.983]	[0.888,0.984]	[0.926,0.990]	[0.991,0.999]	[0.843,0.978]

Twelve patients underwent chest CT scan within one month after surgery to assess changes in tracheal morphology. The two observers demonstrated high consistency in the evaluation of postoperative CT images (Table 2). Therefore, the mean values of all measurements—including diameter, cross-sectional area, and protrusion angle—were used for analysis. The results showed that postoperative NWR for tracheal diameter was 0.73 ± 0.12, preoperative NWR of cross-sectional area was 0.52 ± 0.13, and postoperative tracheal carina angle was 77.11° ± 10.60°.

**Table 2:** Interobserver and intermethod agreement on postoperative CT measurements (*n* = 12).

Parameters	Smallest diameter (mm)	Maximum diameter (mm)	Narrowest cross-sectional area (mm^2^)	Maximum cross-sectional area (mm^2^)	Tracheal carina angle °
Observer1	3.61 ± 0.76	5.19 ± 1.10	13.99 ± 6.51	26.72 ± 10.59	76.73 ± 11.11
Observer2	3.79 ± 0.90	5.25 ± 1.08	14.69 ± 6.21	28.43 ± 9.89	77.50 ± 10.41
ICC	0.954	0.961	0.974	0.969	0.939
95%CI	[0.849,0.987]	[0.872,0.989]	[0.912,0.992]	[0.897,0.991]	[0.802,0.982]

Comparison of these three parameters before and after surgery in these 12 patients (Table 3) showed statistically significant differences (*P* < 0.001). Both the NWR for diameter and cross-sectional area increased postoperatively, while the tracheal carina angle decreased significantly (Table 3).

**Table 3:** Tracheal comparison before and after surgery (*n* = 12).

Parameters	Before surgery	After surgery	*T* value	*P* value	Cohen's d	95%CI
NWR of diameter	0.41 ± 0.17	0.73 ± 0.12	6.463	<0.001	1.8657	[0.1918,0.3899]
NWR of cross-sectional area	0.22 ± 0.19	0.52 ± 0.13	6.451	<0.001	1.8600	[0.2114,0.4303]
Tracheal carina angle °	120.32 ± 10.58	77.11 ± 10.60	19.982	<0.001	5.77	[38.45,47.97]

### 3.4 Radiation dose

The preoperative CT radiation doses in the 17 pediatric patients were as follows: the CT dose index (CTDI), dose-length product (DLP), and effective dose (ED) ranged from 2.14 to 7.25 mGy (4.15 ± 1.34 mGy), 6.07 to 17.16 mGy·cm (10.64 ± 3.43 mGy·cm), and 0.24 to 0.61 mSv (0.37 ± 0.11 mSv), respectively.

The postoperative CT radiation doses in the 12 pediatric patients were as follows: CTDI, DLP, and ED ranged from 1.21 to 7.12 mGy (2.16 ± 1.65 mGy), 17.76 to 64.42 mGy·cm (30.85 ± 16.13 mGy·cm), and 0.69 to 1.74 mSv (1.04 ± 0.42 mSv), respectively.

## 4 Discussion

PAS is a rare congenital cardiovascular malformation, often accompanied by airway stenosis and other cardiac malformations. The condition primarily presents with respiratory symptoms, which can be non-specific, potentially leading to missed or misdiagnosed cases, especially with limited awareness of the disease. Delayed treatment is associated with high mortality, making early diagnosis and appropriate clinical intervention crucial (6, 7).

The clinical manifestations of PAS depend on the degree of compression of the trachea and esophagus and the presence of intracardiac malformations (10). This is consistent with the manifestations of the cases in this study. The patients in this study ranged from 7 days to 47 months (median 5 months). None exhibited significant digestive symptoms; all were referred due to respiratory issues. Preoperative chest CT confirmed pulmonary inflammation in all patients, correlating with their symptoms. Postoperative CT scans of patients with improved respiratory symptoms showed significant reduction in pulmonary inflammation, demonstrating CT's utility in objectively assessing pulmonary infection.

Imaging is the primary method for diagnosing PAS. Echocardiography is non-invasive, radiation-free, and highly reproducible, providing both hemodynamic information and visualization of cardiac and great vessel structures, making it the preferred initial examination. A study on 145 PAS patients by Liu et al. (11) reported a diagnostic accuracy of 98.62% for echocardiography. Despite its high accuracy, misdiagnosis remains possible. Ultrasound poorly visualizes the middle and lower trachea and carina, as acoustic shadowing from the sternum and artifacts from intraluminal air often obscure the view. Moreover, ultrasound cannot clearly depict tracheal morphology or the relationship between mediastinal vessels and the trachea. Therefore, cardiac CTA is recommended following ultrasonography in cases with high suspicion of PAS to improve diagnostic accuracy and evaluate tracheal conditions.

CT plays an irreplaceable role in diagnosing tracheal stenosis, which is currently the preferred method for the diagnosis of tracheal stenosis in outpatient and emergency departments (12, 13). CTA can compensate for potential ultrasound misdiagnosis. It not only demonstrates abnormal cardiac and vascular structures but also clearly shows tracheal morphology, the degree of tracheal stenosis and the relationship between mediastinal vessels and the trachea through post-processing techniques, aiding in PAS classification and pretreatment assessment.

CT imaging offers superior diagnostic visualization (14); however, its primary limitation is the use of ionizing radiation. Children are more sensitive to radiation than adults, owing to their rapid growth and highly active cellular metabolism (15). Hence, minimizing radiation exposure is essential in pediatric CT protocols. In this study, preoperative CT radiation doses for 17 pediatric patients ranged from 2.14 to 7.25 mGy (4.15 ± 1.34 mGy) for CTDI, 6.07 to 17.16 mGy·cm (10.64 ± 3.43 mGy·cm) for DLP, and 0.24 to 0.61 mSv (0.37 ± 0.11 mSv) for ED. For the 12 patients who underwent postoperative CT, the corresponding values were 1.21–7.12 mGy (2.16 ± 1.65 mGy), 17.76–64.42 mGy·cm (30.85 ± 16.13 mGy·cm), and 0.69–1.74 mSv (1.04 ± 0.42 mSv). All values were below diagnostic reference levels reported in the literature (16–19).This demonstrates that we should rationally assess radiation risks. In this study, CT scans were performed in accordance with the principles of justification, optimization, and ALARA (As Low As Reasonably Achievable), enabling diagnostically adequate images at low radiation doses.

Bronchoscopy is considered as the gold standard for diagnosing tracheal stenosis (12). It is invasive, which may provoke tracheal spasm and exacerbate stenosis due to mucosal irritation, and can lead to complications such as gum damage, vocal cord injury, bronchial rupture, and even death. For infants and young children whose smaller tracheal lumens demand greater technical proficiency, bronchoscopy is particularly challenging. To minimize risks, none of the 17 pediatric patients in this study underwent preoperative bronchoscopy; instead, bronchoscopy was deferred until the intraoperative period. Except for 3 patients who declined treatment, bronchoscopy was performed in all 14 remaining patients prior to tracheotomy, confirming the presence of complete tracheal rings. This finding was consistent with preoperative CT scans indicating long-segment tracheal stenosis. Thus, preoperative CT clearly demonstrates the morphology and degree of tracheal narrowing, which is typically sufficient for preoperative planning, and thereby avoiding the risks associated with diagnostic-only preoperative bronchoscopy. Furthermore, no cases of tracheal malacia were observed during intraoperative bronchoscopy, indicating that the tracheal stenosis was not caused by tracheal malacia.

**Figure 1:**
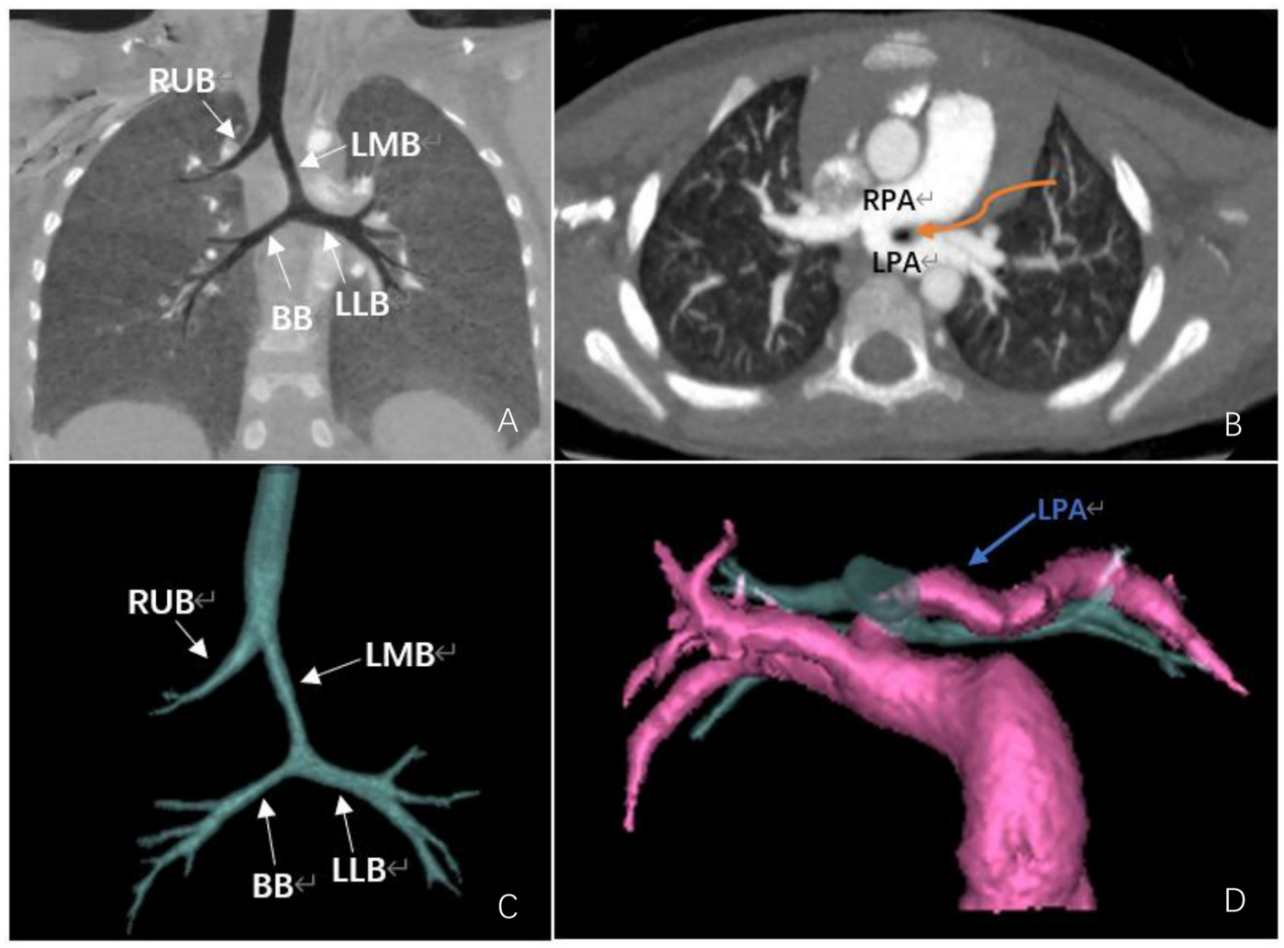
A 2-year-old male; preoperative cardiac CTA suggested pulmonary artery sling type 2A. **(A)** MinIP shows tracheal morphology and confirms the presence of a bronchial bridge. **(B)** Axial MIP showed an abnormal origin of the left pulmonary artery from the right pulmonary artery, with corresponding tracheal compression and narrowing (curved arrow). **(C,D)** Volume rendering and reconstruction showed tracheal morphology and the relationship between the trachea and pulmonary artery. RUB, right upper lobe bronchus; BB, bridging bronchus; LLB, left lower lobe bronchus; LMB, left main bronchus; LPA, left pulmonary artery; RPA, right pulmonary artery.

Preoperative tracheal assessment in this study relied primarily on CT. All 17 patients underwent preoperative cardiac CTA, which identified lung infections and cardiovascular malformations and, through post-processing techniques (20), visualized tracheal morphology and the spatial relationship between the pulmonary artery and trachea (Figure 1), providing surgeons with comprehensive information for optimal treatment planning. Furthermore, tracheal abnormalities can be more objectively assessed through quantification.

Herek et al. (21) reported that the normal tracheal carina angle in children ranges from 80.0° to 88.1°, However, the tracheal carina angle was significantly increased in all children in this study. According to the principle of airflow mechanics, a normal tracheal carina angle may have less resistance (22). Song et al. (23) suggested that tracheoplasty should be performed on patients with a larger tracheal carina angle, which may improve the patient's ventilation function. Additionally, Du et al. (24) reported that for children with the NWR of tracheal diameter ≤0.6, abnormal tracheobronchial branching, long-segment stenosis, and obvious respiratory symptoms, simultaneous tracheal and pulmonary artery sling surgery should be performed, highlighting the safety and efficacy of simultaneous tracheoplasty.

**Figure 2:**
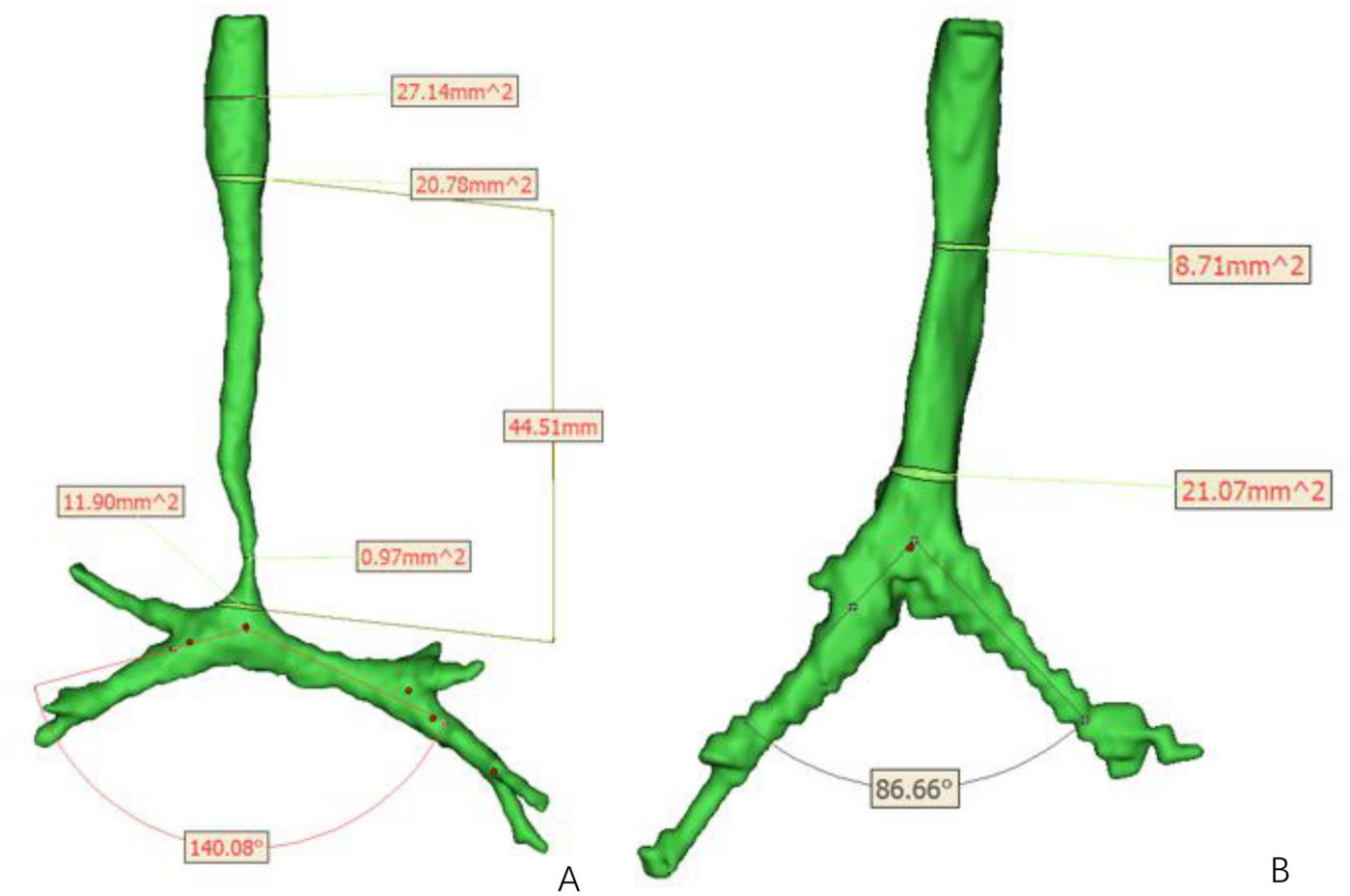
3D reconstruction and measurement of CT images were performed using mimics 21.0. **(A)** Preoperative images showed a long-segment stenosis and an increased carina angle of trachea. **(B)** Postoperatively, the morphology of trachea basically returned normal, and the carina angle decreased. 3D, three-dimensional.

In this study, 14 patients underwent surgical treatment: one underwent staged PAS and tracheal surgery, and 13 underwent simultaneous PAS correction and tracheoplasty. Twelve patients showed significant short-term symptomatic improvement and underwent chest CT follow-up within one month. Comparing preoperative and postoperative tracheal morphology and quantifying the trachea using post-processed CT images, we found that the NWR for both diameter and cross-sectional area increased postoperatively. The tracheal carina angle decreased significantly after surgery (Figure 2). This observation aligns with the report by Du et al. (24).

## 5 Study limitations

This study is a retrospective study and has limitations, including a small sample size and short follow-up period, which preclude assessment of long-term patient prognosis or changes in tracheal morphology during growth and development. Future work should expand the sample size, extend the postoperative follow-up duration, and correlate postoperative evaluations of respiratory and motor function with CT-based quantitative tracheal assessment to better gauge patient health outcomes.

## 6 Conclusions

For patients with prominent respiratory symptoms and suspected of suffering PAS, a combination of echocardiography and cardiac CTA can provide the most accurate diagnosis and avoid missed diagnosis and delayed treatment. With advances in imaging technology and adherence to the principle of radiation protection optimization, chest CT has become a low-dose, non-invasive, and highly efficient diagnostic tool. Both preoperative and postoperative CT are essential for patients with PAS. In cases where preoperative CT suggests long-segment tracheal stenosis, complete tracheal rings should be highly suspected-a finding that can be confirmed intraoperatively via bronchoscopy. Preoperative analysis of the trachea provides crucial support for surgical planning. CT not only detects pulmonary infection but also visualizes tracheal morphological changes before and after surgery via three-dimensional reconstruction and quantitative analysis. This approach enables objective quantification of tracheal morphological improvements, such as an increased tracheal NWR and a reduced carinal angle, thereby reflecting the efficacy of surgery in relieving luminal stenosis and resulting in enhanced tracheal morphology.

## Data Availability

The raw data supporting the conclusions of this article will be made available by the authors, without undue reservation.
